# Association of UDP-galactose-4-epimerase with milk protein concentration in the Chinese Holstein population

**DOI:** 10.5713/ajas.19.0549

**Published:** 2020-02-25

**Authors:** Cong Li, Wentao Cai, Shuli Liu, Chenghao Zhou, Mingyue Cao, Hongwei Yin, Dongxiao Sun, Shengli Zhang, Juan J. Loor

**Affiliations:** 1 Shaanxi Key Laboratory of Molecular Biology for Agriculture, College of Animal Science and Technology, Northwest A&F University, Yangling, Shaanxi 712100, China; 2 College of Animal Science and Technology, Key Laboratory of Animal Genetics and Breeding of Ministry of Agriculture, National Engineering Laboratory for Animal Breeding, China Agricultural University, Beijing 100193, China; 3 Mammalian NutriPhysioGenomics, Department of Animal Sciences and Division of Nutritional Sciences, University of Illinois, Urbana, IL 61801, USA

**Keywords:** Milk Protein Traits, *GALE* Gene, Genetic Effect, Haplotype, Dairy Cattle

## Abstract

**Objective:**

An initial RNA-Sequencing study revealed that UDP-galactose-4-epimerase (*GALE*) was one of the most promising candidates for milk protein concentration in Chinese Holstein cattle. This enzyme catalyzes the interconversion of UDP-galactose and UDP-glucose, an important step in galactose catabolism. To further validate the genetic effect of *GALE* on milk protein traits, genetic variations were identified, and genotypes-phenotypes associations were performed.

**Methods:**

The entire coding region and the 5′-regulatory region (5′-UTR) of *GALE* were re-sequenced using pooled DNA of 17 unrelated sires. Association studies for five milk production traits were performed using a mixed linear animal model with a population encompassing 1,027 Chinese Holstein cows.

**Results:**

A total of three variants in *GALE* were identified, including two novel variants (g.2114 A>G and g.2037 G>A) in the 5′-UTR and one previously reported variant (g.3836 G>C) in an intron. All three single nucleotide polymorphisms (SNPs) were associated with milk yield (p<0.0001), fat yield (p = 0.0006 to <0.0001), protein yield (p = 0.0232 to <0.0001) and protein percentage (p<0.0001), while no significant associations were detected between the SNPs and fat percentage. A strong linkage disequilibrium (D’ = 0.96 to 1.00) was observed among all three SNPs, and a 5 Kb haplotype block involving three main haplotypes with GAG, AGC, and AGG was formed. The results of haplotype association analyses were consistent with the results of single locus association analysis (p<0.0001). The phenotypic variance ratio above 3.00% was observed for milk protein yield that was explained by SNP-g.3836G >C.

**Conclusion:**

Overall, our findings provided new insights into the polymorphic variations in bovine *GALE* gene and their associations with milk protein concentration. The data indicate their potential uses for marker-assisted breeding or genetic selection schemes.

## INTRODUCTION

Milk proteins are important nutrients and milk protein concentration serves as valuable index for milk quality. Dairy industry concerns have driven increasing efforts to improve milk protein concentration [1]. With the development of genomics, bioinformatics and statistical genetics, a single gene or chromosome segments affecting important economic traits can be analyzed [2]. It is possible to improve milk protein concentration through marker assisted selection (MAS) or genomic selection schemes, the challenge, however, is to identify key genes or causal variations affecting milk protein traits [3–6]. Our previously published research has identified that UDP-galactose-4-epimerase (*GALE*) was a strong candidate gene for milk protein traits due to its large differential expression (Log_2_ fold-change = −0.74, *q*-value = 4.41E-03) in mammary tissues of cows with high and low milk protein percentage [7]. In addition, strong interactions were also observed between *GALE* and several other genes such as lactalbumin, alpha (*LALBA*), beta 1,4-galactosyltransferase, polypeptide 1 (*B4GALT1*), and UDP-glucose 6-dehydrogenase (*UGDH*) that play important roles in milk composition synthesis (Supplementary Figure S1) [8–10]. Therefore, based on the biological function and transcriptional effects on milk protein traits, the current study mined to screen the full-length coding regions of the *GALE* gene for single nucleotide polymorphisms (SNPs) and to evaluate the genetic effects of polymorphisms on milk production traits in a large Chinese Holstein population.

## MATERIALS AND METHODS

### Ethics statement

Animal handling and sample collection procedures were performed in accordance with protocols approved by the Institutional Animal Care and Use Committee (IACUC) at China Agricultural University (Permit Number: DK996).

### Genetic sampling

A total of 1,027 Chinese Holstein cows and their 17 corresponding sires were considered as the study population. Cows were selected from 17 farms in the Beijing Sanyuan Lvhe Dairy Farm Center, where routine standard performance test, i.e. Dairy Herd Improvement system (DHI) has been implemented since 1999. The phenotype observations for all individuals for five milk production traits (305 d milk yield, 305 d protein yield, 305 d fat yield, average 305 d protein percentage and average 305 d fat percentage) were collected for subsequent analyses via the complete DHI data from the Chinese dairy cattle population.

### Single nucleotide polymorphism identification and genotyping

Blood samples were collected from 1,027 cows via coccygeal vein to isolate genomic DNA using DP (318) Blood DNA kits (TianGen, Beijing, China). Genomic DNA were also isolated from frozen semen of 17 sires using standard phenol-chloroform procedures and were pooled with 50 ng/μL DNA of each individual to identify variants of the *GALE* gene. *GALE* gene is 4,591 bp in length located at BTA2, contains 11 exons and 10 introns encoding 348 amino acids. All exons and their adjacent intronic sequences were targeted for selective amplification by polymerase chain reaction (PCR). A total of 12 pairs of nucleotide primers (Supplementary Table S1) targeting the regions of interest were designed using Primer3 based on the genomic sequence of the bovine *GALE* gene referring to Bos_taurus_UMD_3.1 assembly (NCBI reference sequence: AC_000159.1).

The PCR amplification was performed in a total volume of 25 μL containing 50 to 100 ng of genomic DNA, 0.5 μL of each primer, 2.5 mM of dNTP mix, 2.5 μL of 10× PCR buffer, and 1 U of Taq DNA Polymerase (Takara Biotechnology Co., Ltd, Dalian, China). The PCR reaction conditions included a pre-denaturation at 95°C for 5 min, followed by 34 cycles of 94°C for 30 s, annealing from 46°C to 56°C for 30 s, 72°C for 30 s, and a final extension at 72°C for 10 min. The PCR products were directly sequenced using the ABI3730xl DNA analyzer (Applied Biosystems, Foster City, CA, USA), and the sequences aligned to the bovine reference sequence (UMD3.1.1) using BLAST (http://blast.ncbi.nlm.nih.gov/Blast.cgi) to identify potential SNPs.

The details of novel SNPs that were identified in the present study were submitted to dbSNP (http://www.ncbi.nlm.nih.gov/SNP/) and are publicly available (accession numbers from ss1996900612 to ss1996900613). All identified SNPs for subsequent genotyping in the 1,027 Chinese Holstein cows were performed with matrix-assisted laser desorption/ionization time of flight mass spectrometry (MALDI-TOF MS, Squenom MassARRAY, Bioyong Technologies Inc. Hong Kong, China) assay.

### Linkage disequilibrium analysis

Haploview [11] was used to measure pairwise linkage disequilibrium (LD) for all identified SNPs within *GALE*. Briefly, missing genotypes were first imputed for each individual using the Beagle3.2 software program [12]. Subsequently, the LD blocks were generated with the subject genotype data using the LD coefficient (D’) [13]. A haplotype with a frequency >5% was considered as a distinguishable haplotype, while the haplotypes with relative frequency <5% were pooled into a single group. Haplotype blocks within these SNPs were used to test their associations with milk production traits.

### Association analyses

A goodness-of-fit test (Chi-square) was applied to compare the numbers of expected and observed genotypes to test Hardy-Weinberg equilibrium for each identified SNP, 0.05 as the significant threshold value. Association analyses were conducted to estimate the effects of *GALE* variants on milk production traits based on both single SNP genotypes and the haplotype blocks. The effects of single SNP or haplotypes in *GALE* on the five milk production traits were analyzed with the mixed procedure of SAS9.3 software (SAS Institute Inc., Cary, NC, USA) using the following mixed linear animal model:

$${\text{y}}_{\text{ijklmn}}=\mu +{\text{F}}_{i}+YS_{j}+P_{k}+\text{b}\times \text{M}+G_{l}+\alpha_{\text{m}}+{\text{e}}_{\text{ijklmn}}$$

where, y_ijklmn_ was the phenotypic value of each trait for each cow (n = 1,027 for each trait); *μ* was the overall mean; *F_i_* was the fixed effect of farm; *YS_j_* was the fixed effect of year-season; *P_k_* was the fixed effect of parity; M was the covariate effect of calving month; b was the regression coefficient of M; *G_l_* was the fixed effect corresponding to the genotype of polymorphisms or haplotype (genotypes of SNPs were modelled as 0–1–2, haplotypes were modelled as 0–5); α_m_ was the random polygenic effect, distributed as N (0, Aσ_a_^2^), with the additive genetic relationship matrix A and the additive genetic variance σ_a_^2^; A-matrix was constructed by tracing the pedigree back to three generations of 2,312 involved individuals; and e_ijklmn_ was the random residual, distributed as N (0, Iσ_e_^2^), with identity matrix I and residual error variance σ_e_^2^.

For single SNP and haplotype analyses, the Bonferroni method was adopted to correct for multiple-testing according to the number of SNP loci or haplotype blocks. Associations were considered as significant if a raw p value <0.05/N, where N was the number of SNP loci or haplotype blocks tested in analyses. The additive (a), dominance (d), and allele substitution (α) effects were estimated using the equation from Falconer and Mackay [14], i.e. a = (AA–BB)⁄2, d = AB–(AA+BB)⁄2, and α = a+d(q–p), where AA and BB represented the two homozygous genotypes, AB was heterozygous genotype, and p and q were the allele frequencies of corresponding loci.

The effect of a SNP on a specific trait was measured as the proportion of phenotypic variance of the trait explained by the SNP. The proportion of variance explained by a SNP was calculated as $2p(1-p)\alpha^{2}/\sigma_{p}^{2}$, where *p* was the allele frequency of SNP, *α* was the average effect of gene substitution calculated based on the linear mixed model, and $\sigma_{p}^{2}$ was the estimate of phenotypic variance using the complete DHI data of the Chinese dairy cattle population.

## RESULTS

A total of three SNPs were discovered in the *GALE* gene of which two (g.2114A>G and g.2037G>A) in the 5′-UTR are novel (ss1996900612 and ss1996900613). The other SNP (g.3836G>C) previously reported was located in the intronic region (rs211659075) (Table 1). All three SNPs were in Hardy-Weinberg equilibrium (p>0.05, Table 2).

The association results between the identified SNPs in *GALE* and five milk production traits are presented in Table 3. All three SNPs (g.2114A>G, g.2037G>A and g.3836G>C) were highly associated with milk yield (p<0.0001), fat yield (p = 0.0006 to <0.0001), protein yield (p = 0.0232 to <0.0001), and protein percentage (p<0.0001). No significant associations were observed between the SNPs and milk fat percentage. Greater than 1% of phenotypic variation accounted for by the three SNPs was detected in six significant SNP-trait pairs. Within these pairs, the pairs of g.3836G>C and milk yield, g.3836G>C and milk protein yield and g.3836G>C and milk protein percentage accounted for up to 2.61%, 3.00%, and 1.08% of phenotypic variation, respectively. In addition, significant additive effects, dominant effects and allele substitution effects were observed for the significant related traits (Table 4).

**Figure 1:**
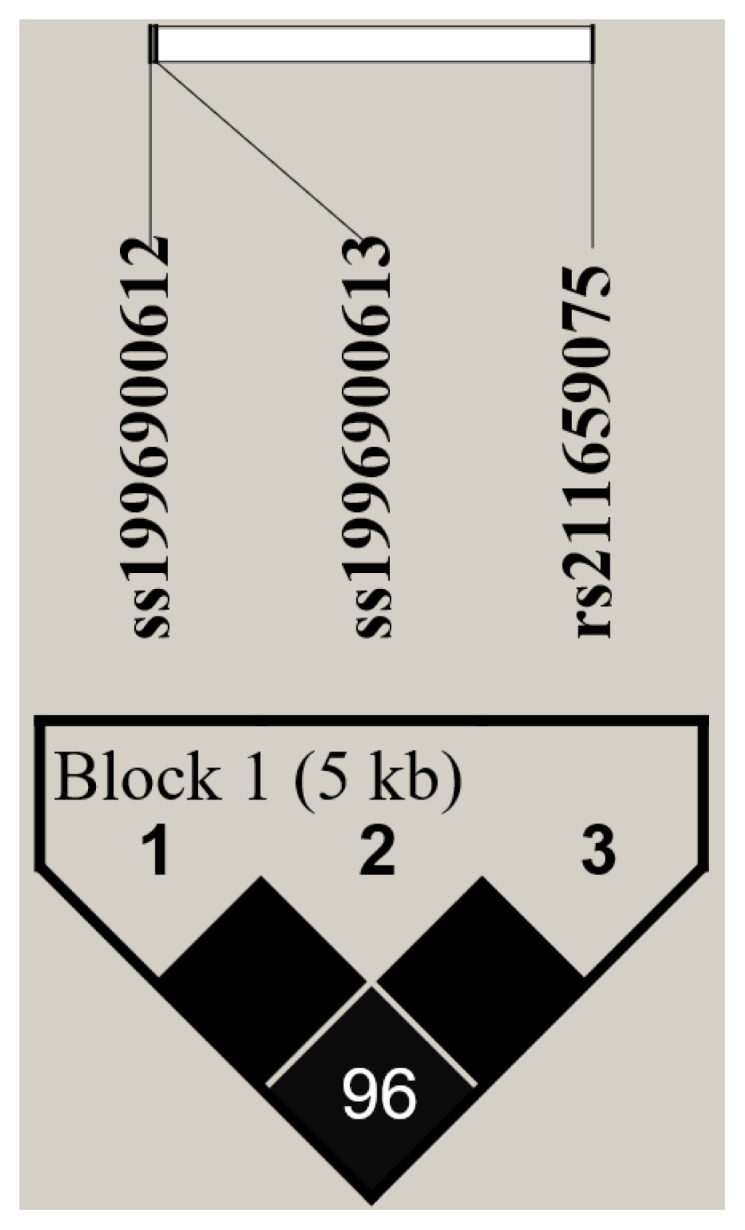
The haplotype blocks and pairwise linkage disequilibrium (LD) values (D’) for the UDP-galactose-4-epimerase (*GALE*) single nucleotide polymorphisms (SNPs). The values within boxes are pair-wise SNP correlation (D’), black boxes without numbers indicate complete LD (D’ = 1).

The specific LD results are showed in Supplementary Table S2–S3 and Supplementary Figure S2. Strong LD (D’ = 0.96 to 1.00) was observed between the three identified SNPs. A 5 Kb haplotype block was inferred (Figure 1), and three major haplotypes were constructed: GAG, AGC and AGG, with frequencies of 59.8%, 19.8%, and 15.0%, respectively (Table 5). A total of six genotypes of haplotypes, H1H1, H2H1, H2H2, H2H3, H3H1, and H3H3 (H1 = GAG, H2 = AGC, H3 = AGG) were formed. Haplotype association analysis revealed consistent results with single-locus analysis (Table 6). The haplotypes H1 and H3 had higher milk yield and protein yield than H2 haplotype (Table 6).

## DISCUSSION

Polymorphisms located in the promoter of a gene may affect transcription by altering transcription factor binding sites or RNA stability [15], which indicated the importance of the two novel SNPs (g.2114A>G and g.2037G>A) identified in the 5′-UTR of *GALE*. The intronic SNP (g.3836G>C) may have a potential regulatory effect on gene expression, regulation, transcription and mRNA splicing, although it does not hold a sequence encoding a protein [16–19]. The greater expression of *GALE* in mammary tissues of cows with high versus low milk protein percentage [7] agreed with such effect. To our knowledge, this was the first evidence showing significant associations of the *GALE* gene with milk protein traits in dairy cattle.

From a statistical standpoint, the single SNP association analysis was less powerful than multiple SNPs analysis due to the lack of simultaneous use of multiple SNPs information [20,21]. Thus, the haplotype-based association analysis was further performed to investigate the association of *GALE* variants with milk production traits in the present study. We observed that the three identified SNPs were associated with milk yield and milk protein traits, which was further confirmed by haplotype-based association analysis. Both single and haplotype association analyses demonstrated that the *GALE* gene was a promising candidate gene affecting milk yield and protein traits.

Protein GALE is UDP-galactose-4-epimerase, which catalyzes the interconversion of UDP-galactose and UDP-glucose in the final step of the Leloir pathway [22,23], and catalyzes the epimerization of UDP-N-acetylglucosamine to UDP-N-acetylgalactosamine [24,25]. *GALE* plays critical roles in dietary galactose metabolism, endogenous galactose production, and glycoprotein and glycolipid biosynthesis [26,27]. String interaction network (https://string-db.org/network/9606.ENSP00000363621) revealed that GALE protein interacts with LALBA, UDP-Gal: betaGlcNAc B4GALT1, and UGDH. Among this list, LALBA is the major component of milk protein and a subunit of lactose synthase. As one of the well-studied glycosyltransferases, B4GALT1 is responsible for the synthesis of complex-type N-liked oligosaccharides in many glycoproteins [8]. The association of polymorphisms of the *B4GALT1* with milk production traits in Holstein cows has been reported in previously published research [9]. The *UGDH* gene was suggested to be associated with milk yield and milk composition [10]. Taken altogether, the influences of *GALE* on milk production and composition are likely due to the interaction of *GALE* with those known genes.

## CONCLUSION

Results in the present study demonstrated the significant genetic effects of *GALE* on milk protein traits, which is in close agreement with our previous RNA-Seq study. Results also confirmed the phenotypes of milk protein were directly affected by *GALE* gene at genome level and transcriptional level. Due to the high phenotypic variance ratio, the SNP g.3836G>C in bovine *GALE* may be the most promising marker implicated in milk protein concentration in dairy cattle and has the capability to be used in MAS. Hence, results lay a preliminary foundation for further identifying the causal mutations affecting milk proteins in follow-up studies.

## Figures and Tables

**Table 1:** Information for the three identified single nucleotide polymorphisms in UDP-galactose-4-epimerase gene

CHR	RefSNP	SNP locus	Alleles	Location	Position	Gene
2	ss1996900612	g.2114A>G	A/G	5′-UTR	129705167	*GALE*
2	ss1996900613	g.2037G>A	G/A	5′-UTR	129705244	*GALE*
2	rs211659075	g.3836G>C	G/C	Intron-9	129711117	*GALE*

SNPs, single nucleotide polymorphisms; *GALE*, UDP-galactose-4-epimerase.

**Table 2:** Genotypic and allelic frequencies and Hardy-Weinberg equilibrium test of three single nucleotide polymorphisms of UDP-galactose-4-epimerase gene in Chinese Holstein cattle

Position	Locus	Genotypes	N	Frequency	Allele	Frequency	Hardy-Weinberg equilibrium χ^2^ test
5′flanking region	ss1996900612	AG	457	0.454	A	0.350	p>0.05
	g.2114A>G	AA	124	0.123	G	0.650	
		GG	425	0.422			
5′flanking region	ss1996900613	AG	491	0.483	A	0.594	p>0.05
	g.2037G>A	AA	358	0.352	G	0.406	
		GG	167	0.164			
Intron-9	rs211659075	CG	326	0.323	C	0.198	p>0.05
	g.3836G>C	CC	37	0.037	G	0.802	
		GG	645	0.640			

**Table 3:** Associations of UDP-galactose-4-epimerase single nucleotide polymorphisms with milk production traits in Chinese Holstein cattle (LSM±SE)

Locus	Genotypes	Milk yield (kg)	Fat yield (kg)	Fat percentage (%)	Protein yield (kg)	Protein percentage (%)
ss 1996900612	AA(124)	10,507±88.95^AB^	371.88±3.75^AB^	3.595±0.036	331.02±2.73^ab^	3.171±0.012^A^
g.2114A>G	AG(457)	10,402±61.90^A^	364.66±2.59^A^	3.585±0.025	327.83±1.89^a^	3.192±0.009^A^
	GG(425)	10,667±63.55^B^	373.54±2.65^B^	3.546±0.026	332.53±1.93^b^	3.136±0.009^B^
	p-value1)	**<0.0001**	**0.0006**	0.1567	**0.0232**	**<0.0001**
	Variance	0.05%	0.13%	0.22%	0.02%	0.16%
ss 1996900613	AA(358)	10,768±65.48^A^	375.79±2.74^A^	3.543±0.026	335.94±1.99^A^	3.141±0.009^A^
g.2037G>A	AG(491)	10,326±61.30^B^	361.05±2.56^B^	3.572±0.025	324.63±1.87^B^	3.185±0.009^B^
	GG(167)	10,404±81.06^B^	368.40±3.40^AB^	3.602±0.033	327.01±2.48^B^	3.169±0.011^B^
	p-value1)	**<0.0001**	**<0.0001**	0.1643	**<0.0001**	**<0.0001**
	Variance	**1.64%**	0.14%	0.50%	**1.03%**	0.30%
rs211659075	CC(37)	9,597.66±141.44^A^	338.99±5.98^A^	3.605±0.057	301.50±4.36^A^	3.166±0.020^AB^
g.3836G>C	CG(326)	10,359±66.80^B^	363.19±2.80^B^	3.596±0.027	326.87±2.04^B^	3.202±0.009^A^
	GG(645)	10,581±59.48^C^	370.71±2.48^C^	3.559±0.024	330.91±1.81^B^	3.154±0.008^B^
	p-value1)	**<0.0001**	**<0.0001**	0.2294	**<0.0001**	**<0.0001**
	Variance	**2.61%**	**1.56%**	0.08%	**3.00%**	**1.08%**

LSM, least square mean; SE, standard error; SNP, single nucleotide polymorphisms.

1) p-value refers to the results of association analysis between each SNP and milk production traits.

Different letter (small letters, p<0.05; capital letters, p<0.01) superscripts (adjusted value after correction for multiple testing) indicate significant differences among the genotypes. $\text{Phenotypic}\;\text{variance}\;\text{ratio}=2p(1-p)\alpha^{2}/\sigma_{p}^{2}$.

**Table 4:** Additive, dominant and allele substitution effects of the three single nucleotide polymorphisms associated with milk production traits of UDP-galactose-4-epimerase in Chinese Holstein

Locus	Genetic effect	Milk yield	Fat yield	Fat percentage	Protein yield	Protein percentage
ss 1996900612	Additive	79.68±42.75	−0.83±1.81	0.0245±0.02	−0.75±1.32	0.0176±0.01**
g.2114A>G	Dominant	−184.83±56.79**	−8.05±2.41**	0.0146±0.02	−3.95±1.76*	0.0384±0.01**
	Substitution	−24.38±51.78	1.58±2.19	0.0201±0.02	0.43±1.60	0.0061±0.01
ss 1996900613	Additive	182.15±39.80**	3.70±1.69*	−0.0295±0.02	4.46±1.23**	−0.0140±0.01**
g.2037G>A	Dominant	−259.98±52.84**	−11.05±2.24**	−0.0001±0.02	−6.84±1.63**	0.0304±0.01**
	Substitution	133.27±43.35**	1.62±1.84	−0.0295±0.02	3.18±1.34*	−0.0083±0.01
rs211659075	Additive	−491.46±69.94**	15.86±2.96**	0.0229±0.03	−14.70±2.16**	0.0062±0.01
g.3836G>C	Dominant	269.59±81.71**	8.34±3.47**	0.0140±0.03	10.66±2.53**	0.0420±0.01**
	Substitution	−654.07±111.31**	−20.89±4.72**	0.0144±0.04	−21.13±3.44**	−0.0191±0.02

* Means differ at p<0.05.

** Means differ at p<0.01.

**Table 5:** Main haplotypes and their frequencies observed in *GALE* gene

GALE haplotypes	ss1996900612 A>G	ss1996900613 G>A	rs211659075 G>C	Frequency (%)
GAG	G	A	G	59.8
AGC	A	G	C	19.8
AGG	A	G	G	15.0

*GALE*, UDP-galactose-4-epimerase; SNPs, single nucleotide polymorphisms.

The Ref number of each SNP can be found in the haplotype Figure 1.

**Table 6:** Haplotype associations of *GALE* single nucleotide polymorphisms with milk production traits in Chinese Holstein cattle (LSM±SE)

GALE haplotypes	Milk yield (kg)	Fat yield (kg)	Fat percentage (%)	Protein yield (kg)	Protein percentage (%)
H1H1(369)	10,779±66.50^A^	378.16±2.78^AC^	3.548±0.027	336.27±2.03^A^	3.140±0.009^A^
H2H1(243)	10,403±72.46^B^	362.51±3.04^BD^	3.558±0.029	328.14±2.21^B^	3.199±0.010^B^
H2H2(39)	9,891.26±138.99^C^	350.80±5.86^B^	3.611±0.056	311.33±4.27^C^	3.172±0.020^AB^
H2H3(61)	10,798±118.84^AD^	387.90±5.01^A^	3.628±0.047	341.15±3.65^A^	3.175±0.016^AB^
H3H1(181)	10,535±79.06^BD^	371.77±3.31^CD^	3.578±0.032	331.72±2.41^AB^	3.177±0.011^B^
H3H3(26)	10,986±173.95^AD^	369.53±7.35^ABC^	3.405±0.070	342.22±5.36^AB^	3.137±0.024^AB^
p-value1)	<0.0001	<0.0001	0.0807	<0.0001	<0.0001

*GALE*, UDP-galactose-4-epimerase; LSM, least square mean; SE, standard error.

1) p-value refers to the results of association analysis between each haplotype and milk production traits.

Different letter superscripts indicate significant differences among the haplotypes (p<0.01). H1, H2, and H3 represented the types of haplotypes, of these, H1 = GAG, H2 = AGC, H3 = AGG.

## References

[1] Dallas DC, Murray NM, Gan JN (2015). Proteolytic systems in milk: perspectives on the evolutionary function within the mammary gland and the infant. J Mammary Gland Biol Neoplasia.

[2] Carta A, Casu S, Salaris S (2009). Invited review: Current state of genetic improvement in dairy sheep. J Dairy Sci.

[3] Chen HY, Zhang Q, Yin CC, Wang CK, Gong WJ, Mei G (2006). Detection of quantitative trait loci affecting milk production traits on bovine chromosome 6 in a Chinese Holstein population by the daughter design. J Dairy Sci.

[4] Gebreyesus G, Lund MS, Janss L (2016). Short communication: Multi-trait estimation of genetic parameters for milk protein composition in the Danish Holstein. J Dairy Sci.

[5] Kolbehdari D, Wang Z, Grant JR (2009). A whole genome scan to map QTL for milk production traits and somatic cell score in Canadian Holstein bulls. J Anim Breed Genet.

[6] Sanchez MP, Ferrand M, Gele M (2017). Short communication: Genetic parameters for milk protein composition predicted using mid-infrared spectroscopy in the French Montbeliarde, Normande, and Holstein dairy cattle breeds. J Dairy Sci.

[7] Li C, Cai W, Zhou C (2016). RNA-Seq reveals 10 novel promising candidate genes affecting milk protein concentration in the Chinese Holstein population. Sci Rep.

[8] Amado M, Almeida R, Schwientek T, Clausen H (1999). Identification and characterization of large galactosyltransferase gene families: galactosyltransferases for all functions. Biochim Biophys Acta Gen Subj.

[9] Shahbazkia HR, Aminlari M, Cravador A (2012). Association of polymorphism of the beta(1, 4)-*galactosyltransferase-I* gene with milk production traits in Holsteins. Mol Biol Rep.

[10] Xu Q, Mei G, Sun DX (2012). Detection of genetic association and functional polymorphisms of *UGDH* affecting milk production trait in Chinese Holstein cattle. BMC Genomics.

[11] Barrett JC, Fry B, Maller J, Daly MJ (2005). Haploview: analysis and visualization of LD and haplotype maps. Bioinformatics.

[12] Browning SR, Browning BL (2007). Rapid and accurate haplotype phasing and missing-data inference for whole-genome association studies by use of localized haplotype clustering. Am J Hum Genet.

[13] Gabriel SB, Schaffner SF, Nguyen H (2002). The structure of haplotype blocks in the human genome. Science.

[14] Falconer DS, Mackay TFC (1996). Introduction to quantitative genetics.

[15] Huang YZ, Li MX, Wang J (2013). A 5′-regulatory region and two coding region polymorphisms modulate promoter activity and gene expression of the growth suppressor gene *ZBED6* in cattle. Plos One.

[16] Nott A, Muslin SH, Moore MJ (2003). A quantitative analysis of intron effects on mammalian gene expression. RNA.

[17] Park HJ, Lee S, Ju E, Jones JA, Choi I (2017). Alternative transcription of sodium/bicarbonate transporter SLC4A7 gene enhanced by single nucleotide polymorphisms. Physiol Genomics.

[18] Sagne C, Marcel V, Amadou A, Hainaut P, Olivier M, Hall J (2013). A meta-analysis of cancer risk associated with the TP53 intron 3 duplication polymorphism (rs17878362): geographic and tumor-specific effects. Cell Death Dis.

[19] Visser M, Palstra RJ, Kayser M (2014). Human skin color is influenced by an intergenic DNA polymorphism regulating transcription of the nearby *BNC2* pigmentation gene. Hum Mol Genet.

[20] Akey J, Jin L, Xiong MM (2001). Haplotypes vs single marker linkage disequilibrium tests: what do we gain?. Eur J Hum Genet.

[21] Martin ER, Lai EH, Gilbert JR (2000). SNPing away at complex diseases: Analysis of single-nucleotide polymorphisms around *APOE* in Alzheimer disease. Am J Hum Genet.

[22] Daenzer JM, Sanders RD, Hang D, Fridovich-Keil JL (2012). UDP-galactose 4′-epimerase activities toward UDP-Gal and UDP-GalNAc play different roles in the development of *Drosophila melanogaster*. PLoS Genet.

[23] Schulz JM, Ross KL, Malmstrom K, Krieger M, Fridovich-Keil JL (2005). Mediators of galactose sensitivity in UDP-galactose 4′-epimerase-impaired mammalian cells. J Biol Chem.

[24] Roper JR, Guther MLS, Milne KG, Ferguson MAJ (2002). Galactose metabolism is essential for the African sleeping sickness parasite *Trypanosoma brucei*. Proc Natl Acad Sci USA.

[25] Seo A, Gulsuner S, Pierce S (2019). Inherited thrombocytopenia associated with mutation of UDP-Galactose-4-Epimerase (*GALE*). Hum Mol Genet.

[26] Sanders RD, Sefton JMI, Moberg KH, Fridovich-Keil JL (2010). UDP-galactose 4′ epimerase (GALE) is essential for development of *Drosophila melanogaster*. Dis Model Mech.

[27] Song HB, He M, Cai ZP (2018). UDP-glucose 4-epimerase and-1,4-galactosyltransferase from the oyster *Magallana gigas* as valuable biocatalysts for the production of galactosylated products. Int J Mol Sci.

