# Supplementary material for: Association of UDP-galactose-4-epimerase with milk protein concentration in the Chinese Holstein population
Source: Asian-Australas J Anim Sci. 2020 Feb 25;33(11):1725–31. doi: 10.5713/ajas.19.0549 (PMC7649079; doi:10.5713/ajas.19.0549)
Supplement: Supplementary file 1 [file ajas-19-0549-suppl.pdf]

Association of UDP-galactose-4-epimerase (*GALE*) with milk protein concentration in Chinese Holstein population

Cong *Li*[1], Wentao *Cai*[2], Shuli *Liu*[2], Chenghao *Zhou*[2], Mingyue *Cao*[2], Hongwei *Yin*[2], Dongxiao *Sun*[2], Shengli *Zhang*[2]*, Juan J. *Loor*[3]*

[1]Shaanxi Key Laboratory of Molecular Biology for Agriculture, College of Animal Science and Technology, Northwest A&F University, Yangling, Shaanxi 712100, China

[2]College of Animal Science and Technology, Key Laboratory of Animal Genetics and Breeding of Ministry of Agriculture, National Engineering Laboratory for Animal Breeding, China Agricultural University, Beijing 100193, China

[3]Mammalian NutriPhysioGenomics, Department of Animal Sciences and Division of Nutritional Sciences, University of Illinois, Urbana, Illinois 61801, United States

Supplementary Files

Table S1 PCR primers information of *GALE* gene

**Primer**	**Location**	**Primer sequences (5’-3’)**	**Product size (bp)**	**Annealing Temp (℃)**
GALE-1	5’ flanking region	F-AAGGAGGTGGACAGGAGTTG<br>R-AAGCCTCCGTTTTCCTCTCA	**517****	59
GALE-2	5’ flanking region	F-AGAGGAAAACGGAGGCTTCA<br>R-ACACGACTGAGCCACTAACA	639	59
GALE-3	5’ flanking region	F-AGTCGTGTCCAGTCGTGTC<br>R-TGTTAGTAAGCCTGGCGTGA	523	59
GALE-4	Exon-1	F-GACCCACTGTTTTCCATCGG<br>R-CCCTCACCAGCTTCCCAAA	533	59
GALE-5	Exon-2	F-ATCTCGTCTGACCCGTACAC	516	59

		R-CAAAATCAGAGGCGATGGGG		
GALE-6	Exon-3/4	F-TCCCCATCGCCTCTGATTTT<br>R-CAGAGGAGAGGTGTCAGTGG	564	59
GALE-7	Exon-5	F-ACACACTGGACACGTCTTCA<br>R-CCAAAAGGCCTGAAGTCACC	444	59
GALE-8	Exon-6/7	F-TCAGGAAGCAGTGGTGACTT<br>R-AGAGGAGGAAGTGGTTGCAG	510	59
GALE-9	Exon-7/8	F-AGGGAGAAGGGAAAGGAAGC<br>R-CCAGGTTGTAGATCTGCCCA	442	59
GALE-10	Exon-9/10	F-AGGGGAGAAGGGAAAAGTGG<br>R-CACATCCTGTCCAGCCCTAG	**515****	59
GALE-11	Exon-10	F-ATTTCTGGAGGGCTGGTCAG<br>R-TCTCTCCCTTTTCTGGTCCG	456	59
GALE-12	Exon-11	F-CCTGGGTGCTTACCTGACC<br>R-TCTGGTCGTGTCCTGGATTC	633	59

Note: **SNP are detected.

Table S2. Basic data quality checks for the markers

#	Name	Position	ObsHET	PredHET	HWpval	%Geno	FamTrio	MendErr	MAF	Alleles	Rating
1	ss1996900612	129705167	0.455	0.455	1.0	99.4	0	0	0.351	G:A	☑
2	ss1996900613	129705244	0.482	0.482	1.0	100	0	0	0.405	A:G	☑
3	rs211659075	129711117	0.325	0.325	0.662	99.3	0	0	0.199	G:C	☑

Note: # is the marker number. **Name** is the marker ID specified. **Position** is the marker position specified. **ObsHET** is the marker's observed heterozygosity. **PredHET** is the marker's predicted heterozygosity (i.e. 2*MAF*(1-MAF)). **HWpval** is the Hardy-Weinberg equilibrium p value, which is the probability that its deviation from H-W equilibrium could be explained by chance. **%Geno** is the percentage of non-missing genotypes for this marker. **FamTrio** is the number of fully genotyped family trios for this marker (0 for datasets with unrelated individuals). **MendErr** is the number of observed Mendelian inheritance errors (0 for datasets with unrelated individuals). **MAF** is the minor allele frequency (using founders only) for this marker. **Alleles** are the major and minor alleles for this marker. **Rating** is checked if the marker passes all the tests and unchecked if it fails one or more tests (highlighted in red).

Table S3. Pairwise measures of LD

L1	L2	D'	LOD	r^2	CIlow	CIhi	Dist	T-int
ss1996900612	ss1996900613	1	299.68	0.792	0.99	1	77	427.55
ss1996900612	rs211659075	0.963	127.87	0.432	0.93	0.99	5950	-
ss1996900613	rs211659075	1	118.77	0.367	0.98	1	5873	246.64

Note: **L1 and L2** are the two loci, referenced by their name or name. **D'** is the value of D prime between the two loci. **LOD** is the log of the likelihood odds ratio, a measure of confidence in the value of D'. **r^2** is the correlation coefficient between the two loci. **CIlow** is 95% confidence lower bound on D'. **CIhi** is the 95% confidence upper bound on D'. **Dist** is the distance (in bases) between the loci, and is only displayed if a marker info file has been loaded. **T-int** is a statistic used by the HapMap Project to measure the completeness of information represented by a set of markers in a region.

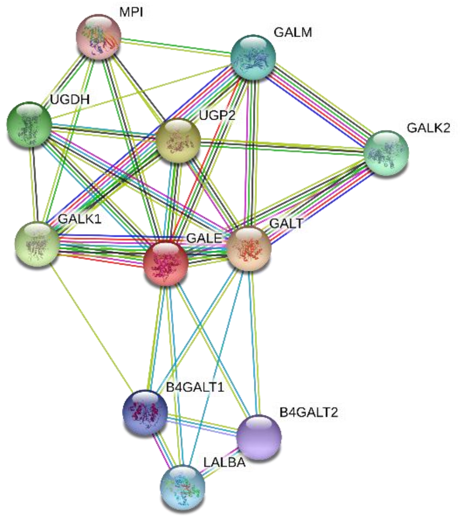

Figure S1. The interaction of GALE with LALBA, B4GALT1 and UGDH

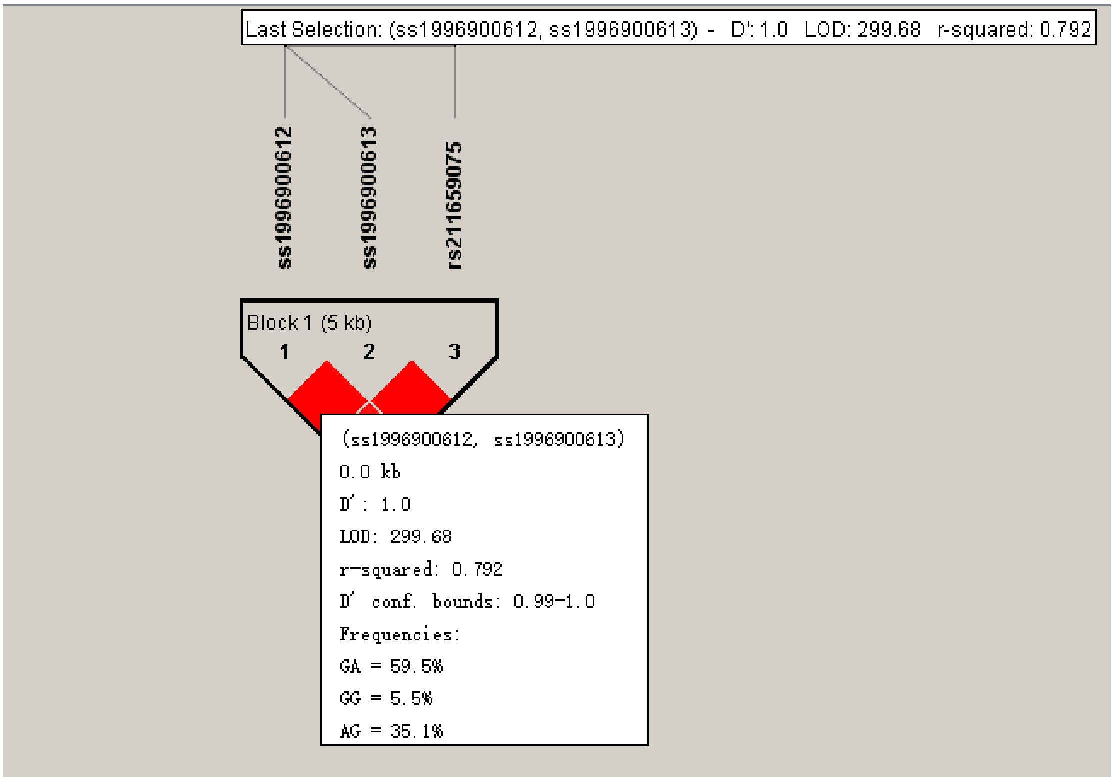

Figure S2. Block 1 comprises marker ss1996900612 and marker ss1996900613

Legend: The white box shows the correlation between marker ss1996900612 and marker ss1996900613 of haplotype block, D' = 1, r^2 = 0.792.
